# *Staphylococcus aureus* Detection in Milk Using a Thickness Shear Mode Acoustic Aptasensor with an Antifouling Probe Linker

**DOI:** 10.3390/bios13060614

**Published:** 2023-06-03

**Authors:** Sandro Spagnolo, Katharina Davoudian, Brian De La Franier, Tibor Hianik, Michael Thompson

**Affiliations:** 1 Faculty of Mathematics, Physics and Informatics, Comenius University, Mlynská Dolina F1, 84248 Bratislava, Slovakia; 2 Department of Chemistry, University of Toronto, 80 St. George Street, Toronto, ON M5S3H6, Canada

**Keywords:** *S. aureus*, antifouling linker, DNA aptamer, thickness shear mode, biosensor, milk

## Abstract

Contamination of food by pathogens can pose a serious risk to health. Therefore, monitoring for the presence of pathogens is critical to identify and regulate microbiological contamination of food. In this work, an aptasensor based on a thickness shear mode acoustic method (TSM) with dissipation monitoring was developed to detect and quantify *Staphylococcus aureus* directly in whole UHT cow’s milk. The frequency variation and dissipation data demonstrated the correct immobilization of the components. The analysis of viscoelastic properties suggests that DNA aptamers bind to the surface in a non-dense manner, which favors the binding with bacteria. The aptasensor demonstrated high sensitivity and was able to detect *S. aureus* in milk with a 33 CFU/mL limit of detection. Analysis was successful in milk due to the sensor’s antifouling properties, which is based on 3-dithiothreitol propanoic acid (DTT_COOH_) antifouling thiol linker. Compared to bare and modified (dithiothreitol (DTT), 11-mercaptoundecanoic acid (MUA), and 1-undecanethiol (UDT)) quartz crystals, the sensitivity of the sensor’s antifouling in milk improved by about 82–96%. The excellent sensitivity and ability to detect and quantify *S. aureus* in whole UHT cow’s milk demonstrates that the system is applicable for rapid and efficient analysis of milk safety.

## 1. Introduction

Nutrition is a fundamental process in human life as it allows the body to obtain nutrients necessary for growth and survival. However, food can pose a risk to human health such as through its potential contamination with pathogens [1]. Milk is no exception; each step of its processing, from milking to distribution, can be affected by microbiological contaminants. Monitoring food for the presence of pathogens is important for identifying and regulating such contamination [2].

There are numerous bacteria that can be found in milk depending on different handling processes of food [2]. Immediately after milking, microorganisms in the milk come largely from environmental contamination and their concentrations can change during processing and manipulation [2]. There are many dangerous pathogenic bacteria capable of growing in milk and dairy products: in particular, those of the genus Campylobacter, Listeria, Salmonella, Brucella, Mycobacterium, Staphylococcus, Clostridium, Bacillus, and Pseudomonas.

*Staphylococcus aureus* (*S. aureus*) bacteria and their toxins can cause serious infections such as sepsis. *S. aureus* bacteria can be found in the environment, such as in dust or soil, and on living organisms. As the bacteria is present in normal human flora, individuals can also be a contamination source for food [3]. The low acidity and high protein content of milk provides an ideal environment for the rapid growth of *S. aureus*. Although cooking milk to high temperatures can kill bacteria, this does not guarantee product safety as any bacterial toxins are not affected. For *S. aureus* regulation in milk, both the bacteria and its toxins must be monitored to determine if their presence exceeds safe limits [4]. Generally, bacteria in milk are detected by growing bacteria using agar plates and counting the number of colonies, which is a slow, overnight process that relies on accurate plate counting [4]. Much faster methods of detection include PCR (polymerase chain reaction), ELISA (Enzyme-linked immunosorbent assay), or ELFA (Enzyme-linked fluorescent assay). Immunoassays are more rapid, lower cost, and simpler. Therefore, immunoassays can be used prior to more detailed analyses by specific PCR. The specificity of PCR is based on detection of the gene related to the bacteria. The sensitivity of the advanced PCR test is 5 CFU/mL in the 20 min [5]. The DNA-based nanobarcode test is also rather rapid [6]. However, the above-mentioned conventional methods require specialized microbial laboratories and well-trained personnel. At the same time, for fast monitoring of food safety, rapid and easy-to-use methods are required. Biosensor technology can fulfill these conditions. 

Biosensors have quickly developed in the last few decades with the progress of materials and electronics engineering, as well as the advent of biochemical and molecular methods for producing probes that selectively bind analytes. New trends are focused on the application of DNA aptamers as receptors that can selectively bind bacteria [7]. DNA aptamers are single-stranded oligonucleotides developed by combinatorial chemistry known as SELEX (Systematic Evolution of Ligands by EXponential enrichment). This method was originally developed for detecting small molecules or proteins in a solution. However, the modified Cell-SELEX method can be used for the development of aptamers for proteins or lipopolysaccharides incorporated in bacterial membranes [8]. Using this method, the aptamers that selectively bind various bacteria have been developed [7]. DNA aptamers are also known as chemical antibodies due to their in vitro selection. However, in contrast with conventional monoclonal antibodies, they are more stable and can be synthesized with high precision. They can be modified by various linkers that allow their immobilization at various surfaces. Among numerous electrochemical or optical aptasensors, those based on mass detection are of substantial interest. The advantage of these acoustics sensors, mostly based on the thickness shear mode method (TSM) and surface acoustic waves (SAW), is label-free detection. Several examples of bacterial acoustics aptasensors have been published and recently reviewed [9].

A very important aspect of biosensor analysis is the ability to carry out the detection of analytes directly on raw samples, which have not been previously treated. However, raw samples can limit detection due to the heterogeneity of biological liquids which can cause significant fouling of the sensing surface. As a result, antifouling chemistry is an important field for targeting the non-specific adsorption (NSA) of biological fluids. A strategy to achieve these intentions is to coat the sensor surface with a layer capable of limiting NSA. Some antifouling molecules work by incorporating interfacial water molecules within and/or on the surface of the layer, effectively creating a “water barrier” that makes fouling thermodynamically unfavorable [10].

Antifouling thiol- or sulfide-based molecules are used for gold electrode sensors, as a self-assembled monolayer (SAM) can be immobilized through gold–sulfur surface chemistry. These molecules include zwitterions, spacers, and derivatives of poly- and oligoethylene glycol. In the literature, different works describe the synthesis of new molecules whose SAM layer can have antifouling properties [11,12]. In our recent work, we described the synthesis of a molecule, 3-dithiothreitol propanoic acid (DTT_COOH_,), with both antifouling and linker properties, i.e., with the ability to prevent NSA and to behave as a molecule capable of anchoring a probe on the sensor surface for specific recognition of the analyte. However, these properties have only been demonstrated in human serum [12]. This approach has been further exploited for the detection of *E. coli* in raw milk using electromagnetic piezoelectric sensor (EMPAS) [10]. This method allowed us to detect bacteria in raw milk with the LOD of 8 CFU/mL. However, EMPAS does not allow analysis of the viscoelastic contribution in bacteria–aptamer interactions. Therefore, in this work, we applied TSM with dissipation monitoring that provides information about dissipation, which is the energy that is lost due to variation in a viscoelastic layer. Dissipation is proportional to the damping of the resonance. As damping increases, more energy is dissipated, which indicates that a more viscoelastic layer has been adsorbed on the surface. For preparation of the aptasensor sensitive to *Staphylococcus aureus*, we used the same linker as in the previous work, namely, DTT_COOH_ [10]. We also analyzed DTT_COOH_ by Fourier transform infrared spectroscopy (FTIR) for functional group and structure confirmation. However, to improve the antifouling properties of the sensing layer, the aptasensor’s SAM layer also included 2-(2-mercaptoethoxy)ethan-1-ol (HS-MEG-OH), a previously synthesized antifouling molecule [13]. HS-MEG-OH was used instead of β-mercaptoethanol as its ether moiety allows for the incorporation of interfacial water molecules. In addition, we also analyzed the viscoelastic properties of the aptamer-based sensing layers. 

## 2. Materials and Methods

### 2.1. Materials

**Figure 1:**
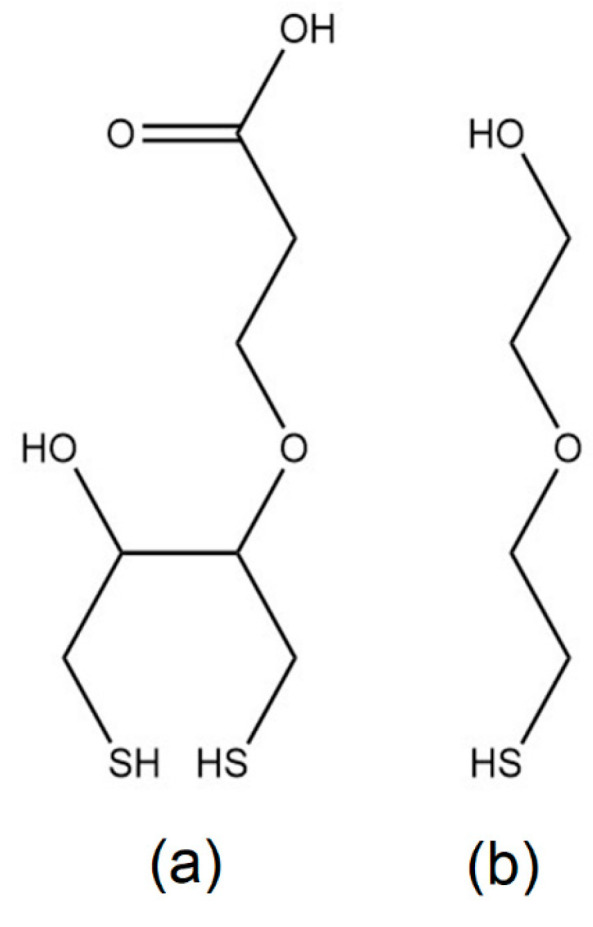
Antifouling thiols used in the experiments: (**a**) 3-dithiothreitol propanoic acid (DTT_COOH_) and (**b**) 2-(2-mercaptoethoxy)ethan-1-ol (HS-MEG-OH).

The synthesis of 3-dithiothreitol propanoic acid (DTT_COOH_) and HS-MEG-OH (Figure 1) followed previously published methods [12,14]. The functional groups of DTT_COOH_ were analyzed by FTIR (see Supplementary Material, Figures S1 and S2). Sodium chloride, absolute analytical grade ethanol, hydrogen peroxide (30% in water *w*/*w*), 1-undecanethiol (UDT), 11-mercaptoundecanoic acid (MUA), dithiothreitol (DTT), N-hydroxysuccinimide (NHS), 1-(3-(dimethylamino)propyl)-3-ethylcarbodiimide hydrochloride (EDC), and ethanolamine were purchased from Sigma-Aldrich (St. Louis, MO, USA). Milli-Q water (specific resistance of 18.2 MΩ∙cm) was used for preparing aqueous solutions. Ethanol was purchased from Caledon Laboratory Chemicals (Georgetown, ON, Canada). The chemicals were used without purifying.

The lyophilized aptamer was purchased from Generi Biotech (Hradec Králové, Czech Republic): 5′ NH_2_-TCC CTA CGG CGC TAA CCT CCC AAC CGC TCC ACC CTG CCT CCG CCT CGC CAC CGT GCT ACA AC-3′ (Mw = 19,076 Da). The aptamer’s sequence follows the work by Chang et al. [15]. The aptamer was resuspended in DNase-free TE buffer (10 mM Tris-HCl and 1 mM EDTA at pH 8.0). The stock solution was diluted with PBS buffer to a final concentration of 10 μM then separated into 100 μL aliquots and stored at −20 °C. The secondary structure of DNA aptamers has been analyzed by OligoAnalyzer Tool^TM^ (Integrated DNA Technologies, Inc., Coralville, IO, USA) and is presented in Supplementary Material (Figure S3).

The preparation of phosphate-buffered saline (PBS) involved 137 mM NaCl, 2.7 mM KCl, 10 mM Na_2_HPO_4_, and 1.8 mM KH_2_PO_4_ at pH 7.4. The buffer was filtered with a 0.22 μm membrane (Merck-Millipore, Darmstadt, Germany). *Staphylococcus aureus* KR3 was purchased from the University of Toronto Medstore (Toronto, ON, Canada). Specificity experiments were carried out with *Escherichia coli* DH5α and *Pseudomonas aeruginosa* PAO1. Whole UHT cow milk (3.5% fat) was bought from Walmart (Toronto, ON, Canada).

### 2.2. Cleaning and Surface Modification of Piezocrystals 

The AT-cut quartz crystals (0.2 cm^2^ sensing area, 8 MHz fundamental frequency) were purchased from Total Frequency Control Ltd., Storrington, UK. The crystals had gold electrodes deposited on both sides. Basic Piranha solution (7 mL of 1:1:5 *v*/*v* 28–30% NH_4_OH, 30% H_2_O_2_, Milli-Q water at 70 °C) was used to clean each crystal in three 25 min cycles. In between the cycles, the crystals were rinsed with Milli-Q water three times. After the third Piranha cycle, the crystals were rinsed with Milli-Q water twice, then with methanol twice. 

**Figure 2:**
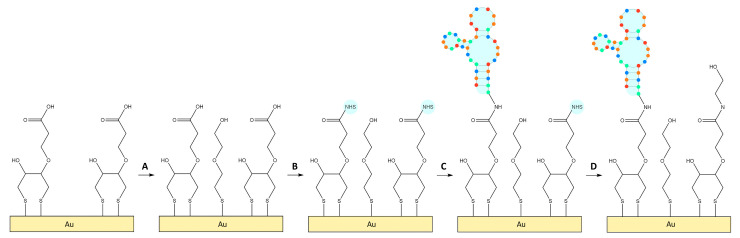
Surface modification of TSM crystals. Following DTT_COOH_. The crystals were functionalized with (A) HS-MEG-OH, then (B) NHS/EDC, (C) aptamer, and (D) ethanolamine. Different colors in aptamer structure mean various nucleotides.

After cleaning, the quartz crystals were functionalized in a solution of 2 mM UDT, 2 mM MUA, 50 µM DTT, or 50 µM DTT_COOH_ in absolute ethanol overnight. For DTT_COOH_-coated crystals, the surfaces were further modified in 2 mM HS-MEG-OH in absolute ethanol (25 min), 20 mM NHS and 50 mM EDC in Milli-Q water (35 min), 5 μM aptamer in Milli-Q water (90 min), and 0.1 M ethanolamine in Milli-Q water (40 min). The crystals were rinsed with Milli-Q water after each solution and dried gently under nitrogen before measurements. For the aptasensor, some crystals were functionalized in-flow to determine if in-vial functionalization was effective (Figure 2).

### 2.3. Contact Angle Goniometry

Static contact angle goniometry (CAG) experiments were conducted with the KSV CAM 101 goniometer (KSV Instruments Ltd., Helsinki, Finland). A 5 µL drop of Milli-Q water at room temperature was used for each measurement. Bare and coated TSM crystals were analyzed in triplicate.

### 2.4. Bacteria Preparation

Lysogeny broth was used to grow *S. aureus* bacteria at 37 °C overnight. The grown solution was serially diluted from 1/10 to 1/10^9^ times in PBS. Each solution was spotted onto agar plates (3 × 10 μL), as well as measured using a UV-1600PC spectrometer (VWR International, Mississauga, ON, Canada) to measure the optical density at 600 nm (OD600). The plates were incubated overnight at 37 °C and spot counted to calculate CFU per OD600 (see Supplementary Material, Section S3).

For TSM measurements, *S. aureus*, *E. coli*, or *P. aeruginosa* bacteria was grown overnight at 37 °C. The next day, 1 mL of the bacteria solution was centrifuged at 14,500× *g* RPM (5 min). A total of 1 mL of PBS was used to resuspend the bacteria pellet. The OD600 of the solution was used to calculate the base CFU, and then the solution was diluted to the desired CFU. To prepare milk samples, the diluted PBS bacteria solution was centrifuged at 14,500× *g* RPM (5 min) to pellet the bacteria, and then the bacteria was resuspended in milk.

### 2.5. TSM Measurements

Cleaned or modified crystals were inserted in an acryl flow-through cell (JKU, Linz, Austria), which was clamped by a holder, ensuring the internal conductors were in contact with the crystal’s electrodes. Liquid flowed through the internal chamber using a GeniePlus syringe pump (Kent Scientific, Torrington, CT, USA) and a pulling syringe. The liquid flowing through the internal chamber was in contact with one face of the crystal. A SARK-110 vector analyzer (Seeed, Shenzhen, China) was used to collect data via Python software [16]. Experiments were measured at 8 MHz under ambient conditions and under a 50 μL∙min^−1^ constant flow. The scheme of experimental setup is presented in Supplementary Material (Figure S5).

Marketed whole milk was used as a fouling agent for antifouling experiments. The crystals were first rinsed with PBS buffer to wash off weakly adsorbed thiol molecules and to reach a stable baseline (about 50 min). After baseline was achieved, the solution was changed to milk samples for bare, UDT, MUA, DTT, and DTT_COOH_ crystals (250 μL over 5 min of flow) then returned to flow under PBS buffer.

For the aptasensor, DTT_COOH_-modified crystals were functionalized according to the same procedure described above (Section 2.2). The crystals were exposed to HS-MEG-OH (25 min), rinsed with PBS (5 min), activated with NHS/EDC (35 min), rinsed with PBS (5 min), incubated with aptamer solution (90 min), rinsed with PBS (5 min), incubated with ethanolamine solution (40 min), then washed with PBS (at least 15 min). HS-MEG-OH was used to functionalize any possible exposed gold on the crystal surface, while ethanolamine neutralized activated carboxyl groups that did not react with aptamer. Once the final PBS wash reached a stable baseline following the aptasensor functionalization, whole milk was flowed (250 μL over 5 min of flow) to analyze the crystal’s antifouling character. Alternatively, for *S. aureus* detection, 250 μL of milk sample containing a known concentration of bacteria (10^2^, 10^3^, 10^4^, 10^5^, 10^6^, or 10^7^ CFU/mL) was poured over the crystal surface. After milk (with or without bacteria), PBS buffer was poured over the crystals to wash them, remove any remaining sample on the surface, and reach a final stable baseline. Each experiment was repeated three times.

### 2.6. TSM Data Analysis

By detecting variations in frequency and dissipation, it is possible to obtain information on the mass deposited on the electrode of the crystal using the Sauerbrey equation [17]:

(1) $$∆f=\frac{-2nf_{0}^{2}}{\sqrt{\rho_{q}{µ}_{q}}}\frac{∆m}{A}.$$

Δ*f* (Hz) is the frequency change, *n* is the number of harmonics, and *f_0_* is the fundamental frequency. Δ*m* is the mass adsorbed on the surface, *A* is the active area of the quartz crystal electrode, *μ_q_* is the shear modulus, 2.947 × 10^10^ Pa, and *ρ_q_* is the density, 2.648 × 10^3^ kg m^−3^, of quartz.

The sensor sensitivity has been evaluated by determination of the limit of detection (LOD) according to [18] as follows:

LOD = LOB + 1.645 SD_lc_, (2)

where SD_lc_ is the standard deviation at low concentration of the sample, and the limit of blank (LOB) is the highest apparent analyte concentration:

LOB = mean blank + 1.645(SD_blank_). (3)

The limit of quantification (LOQ) has been calculated according to the equation:

LOQ = 10 × LOD/3.3.(4)

It is also possible to analyze changes in the viscoelastic properties following the functionalization of the thiol SAM with the aptamer in order to study the density of the bioreceptor anchored on the surface and confirm the success of the functionalization. In particular, the shear modulus, *μ_A_*, the viscosity coefficient, *η_A_*, and the penetration depth, *Γ_A_*, of the evanescent acoustic after the aptamer incubation wave were calculated. To perform this analysis, the Voinova–Voigt viscoelastic model was employed [19]:

(5) $$\Delta f\approx -\frac{h_{1}\rho_{1}\omega }{2\pi \rho_{0}h_{0}}\left(1+\frac{h_{1}^{2}\chi }{3\Gamma^{2}\left(1+\chi^{2}\right)}\right)$$

(6) $$\Delta D\approx \frac{2h_{1}^{3}\rho_{1}\omega }{3\pi f_{0}\rho_{0}h_{0}}\frac{1}{\Gamma^{2}\left(1+\chi^{2}\right)},$$

where 𝜒 = $\frac{\mu_{1}}{\eta_{1}\omega }$; *Γ* is the penetration depth of the shear wave in the liquid medium; $\rho_{0}$ is the density of quartz; $h_{0}$ is the thickness of the quartz crystal; $h_{1}$, $\mu_{1}$, $\eta_{1}$, and $\rho_{1}$ are the thickness, the shear elastic modulus, the viscosity, and the density of the adsorbed film, respectively; and *ω* = 2π*f* is the angular frequency of oscillation. DNA has an average density of 1.7 g × cm^−3^, and the thickness can be obtained by dividing the calculated mass per unit area by the density. 

A Python code based on Yoon et al.’s equation [20] was used to analyze the data. Excel Office 365 (Microsoft Corporation, Albany, NY, USA) and OriginPro 8 (OriginLab Corporation, Northampton, MA, USA) were then used to plot and statistically process the data. The aptasensor was incubated with various concentrations of *S. aureus* in triplicate. 

## 3. Results and Discussion

### 3.1. Contact Angle Goniometry 

Different SAM functionalizations of the gold electrode surfaces were confirmed with CAG. The same values were obtained as the previous work: approximately 55°, 105°, 48°, 43°, and 35° for bare gold, and UDT-, MUA-, DTT-, and DTT_COOH_-modified crystals, respectively [12]. As MUA is hydrophilic, its wettability is higher compared to UDT and bare crystals [12]. UDT crystals showed low wettability compared to bare crystals as UDT is non-polar. The contact angle of DTT was similar to our previous work, as well as to that in the literature [12,21], which confirmed that the crystal was successfully functionalized as DTT-modified TSM discs are more polar than bare gold. As DTT_COOH_ is a derivative of DTT, it was expected that DTT_COOH_-modified surfaces would also be hydrophilic, which was confirmed with CAG. As DTT_COOH_ has a carboxylic acid group, this made DTT_COOH_ SAMs more wettable compared to DTT SAMs.

As DTT SAMs have disordered binding to gold, DTT_COOH_ likely experiences similar binding [22]. Such less dense SAMs can form vacancy islands due to rearranging gold atoms [23]. As a result, we used SH-MEG-OH as an antifouling linear thiol to functionalize any exposed areas of the gold surface. 

### 3.2. Milk Antifouling Test with TSM Method

**Figure 3:**
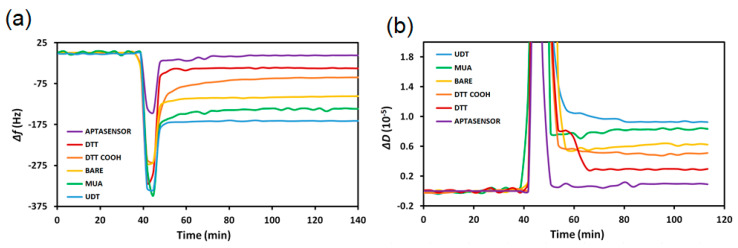
The (**a**) frequency and (**b**) dissipation variations as a result of exposing milk to various SAM-modified quartz crystals.

To determine the aptasensor’s antifouling ability, different SAM-modified quartz crystals were exposed to marketed whole milk (Figure 3). As whole milk contains high protein and fat content, it strongly adsorbs to nonpolar surfaces such as MUA and UDT SAMs. MUA, which is often used as a thiol linker on gold surfaces, experienced slightly less fouling compared to the more hydrophobic UDT SAM crystals. 

As Table 1 summarizes, UDT had the most fouling (158 ± 16 Hz and (8.0 ± 2.4) × 10^−6^ frequency and dissipation shifts, respectively) as its structure is only hydrophobic, while MUA’s hydrocarbon chain and carboxylic acid functional group caused slightly less fouling (136 ± 4 Hz and (7.6 ± 0.8) × 10^−6^ frequency and dissipation shifts, respectively). Bare gold experienced high milk adsorption (105 ± 5 Hz and (6.1 ± 0.6) × 10^−6^ frequency and dissipation shifts, respectively), which is less than for UDT- and MUA-functionalized discs. UDT, MUA, and bare gold demonstrated 64%, 58%, and 45% more fouling relative to DTT_COOH_, respectively.

In comparison, DTT and the aptasensor were, respectively, 31% and 88% less fouling than DTT_COOH_-modified TSM crystals. DTT is likely less fouling (40 ± 8 Hz and (2.8 ± 1.4) × 10^−6^ frequency and dissipation shifts, respectively) than DTT_COOH_ as it does not have a carboxylic acid group that can become negatively charged; instead the DTT SAM remains neutral in milk.

The aptasensor was significantly less fouling compared to DTT_COOH_ as the carboxylic acids are likely deprotonated when exposed to the slightly acidic milk environment. The negatively charged DTT_COOH_ SAM can electrostatically interact with positively charged species, which causes adsorption of milk (58 ± 14 Hz and (3.4 ± 2.7) × 10^−6^ frequency and dissipation shifts, respectively). As the aptasensor’s carboxylic acid groups are modified with aptamers or ethanolamine, the lack of exposed carboxylic acid groups significantly decreases fouling to a minimal amount (7 ± 1 Hz and (0.9 ± 0.2) × 10^−6^ frequency and dissipation shifts, respectively). Modifying the DTT_COOH_ SAM to an aptasensor significantly improves the layer’s antifouling properties, as the neutral and polar surface is likely more effective for interacting with water molecules. Furthermore, the dithiol structure of DTT_COOH_ provides spacing for the water molecules to hydrogen bond with the layer, creating a thermodynamically favorable “water barrier” and largely preventing milk components from adsorbing. Compared to DTT, bare gold, MUA, and UDT crystals, the aptasensor’s fouling significantly decreased by 82%, 94%, 95%, and 96%, respectively. 

### 3.3. Sensing of Staphylococcus Aureus in Milk

**Figure 4:**
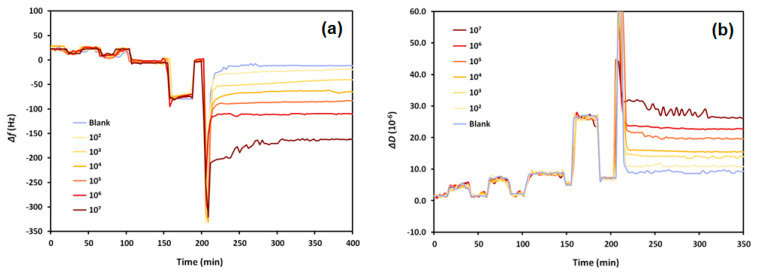
The (**a**) frequency, Δ*f*, and (**b**) dissipation, Δ*D*, shifts as a result of exposing aptasensors to UHT cow’s milk spiked with various concentrations of *S. aureus* in CFU/mL (see the legend).

The aptasensor showed high sensitivity to *S. aureus* in milk. Different bacteria concentrations caused proportional decreases in the resonant frequency. The dissipation changes confirmed the sensitivity as they increased with increasing bacteria concentrations, indicating that *S. aureus* adsorbed to the crystal’s surface by binding with the aptamer. As Figure 4 shows, the crystal experiences minimal frequency and dissipation shifts when exposed to milk without bacteria (8.9 ± 3.4 Hz and (2.1 ± 0.8) × 10^−6^ frequency and dissipation shifts, respectively). Bacteria concentrations in milk were proportional to changes in the frequency and dissipation; increasing cell concentration caused decreasing frequency and increasing dissipation shifts.

**Figure 5:**
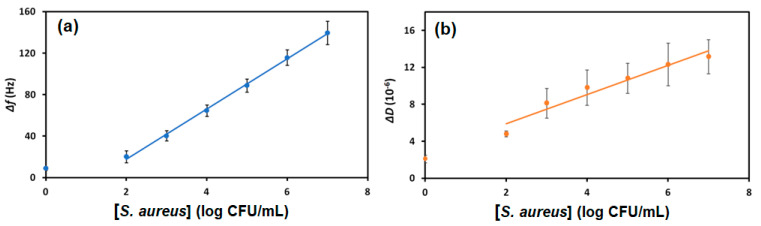
Logarithmic scales with linear fits of (**a**) frequency, Δ*f*, and (**b**) dissipation, Δ*D*, shifts as a result of exposing aptasensors to UHT cow’s milk spiked with various concentrations of *S. aureus.* The equations of the fits are Δ*f* (Hz) = 23.99 log (*S. aureus*)—30.16, R^2^ = 0.999; and Δ*D* = 1.87 log (*S. aureus*) + 1.12, R^2^ = 0.984.

Figure 5 illustrates the proportionality of the changes in frequency and dissipation due to increasing *S. aureus* concentrations in milk. The logarithmic trend indicates that quantification of the bacteria is possible, particularly from measuring frequency changes. The frequency and dissipation variations were calculated from the differences of the stable baselines (before and after sample exposure).

For all concentrations of bacteria in milk tested, a change of approximately 8.9 Hz occurred, which is the mean blank. The standard deviation of the blank (SD_blank_) is 3.4 Hz; therefore, the LOB = 14.5. The SD_lc_ of the low concentration sample is 11.49 Hz; therefore, the LOD was found to be 33.4 CFU/mL. The sensor also showed a dynamic range of 10^2^ to 10^6^ CFU/mL, allowing it to quantify *S. aureus* in a wide range of concentrations.

According to the US Food and Drug Administration (FDA), a concentration of *S. aureus* greater than 10^4^ CFU/mL is considered injurious to health [24]. As this amount is within the dynamic range of the developed sensor and well above the calculated limit of detection, this aptasensor is practical for monitoring milk contamination. The limit of quantification (LOQ) has been calculated according to Equation (4) as 101.2 CFU/mL.

**Figure 6:**
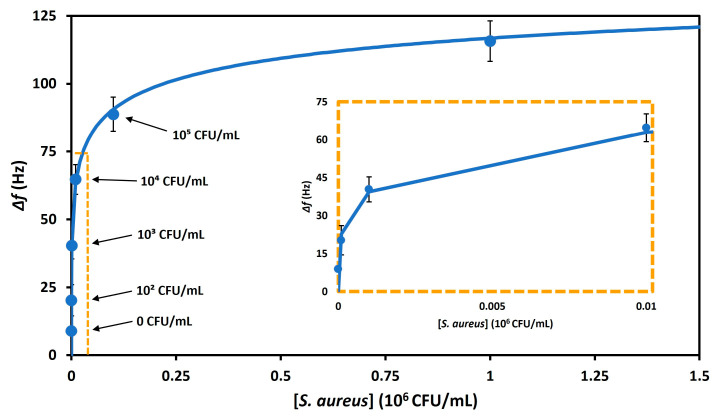
The Langmuir isotherm fit for the frequency shifts vs. *S. aureus* in milk.

Bacteria–aptamer binding was analyzed by fitting the data to the Langmuir isotherm [25] (Figure 6):

Δ*f* = (Δ*f*)_max_ [c/(*K_d_* + *c*)], (7)

where the maximal frequency change is (Δ*f*)_max_, the dissociation constant is *K_d_*, and bacteria concentration is *c*. As the *K_d_* decreases, the binding strength of bacteria–aptamer increases. The calculated *K_d_* and (Δ*f*)_max_ values for bacteria–aptamer binding in whole milk were found to be 270.9 ± 42.9 CFU/mL and 9.8 ± 3.5 kHz, respectively.

Thus, the binding of bacteria to the aptamers resulted in a significant decrease in the frequency and increase in dissipation, which is evident of the viscosity contribution. In this case, the Sauerbrey equation cannot be directly applied for evaluation of the changes of the mass. However, certain rough estimation of the surface density of the aptamers and bacteria can be obtained. According to our data the changes of the frequency following immobilization of aptamers on the antifouling linker can be denoted by Δ*f* = −21.51 ± 3.79 Hz, and corresponding changes in dissipation are Δ*D* = (3.94 ± 0.52) × 10^−6^. If we consider an aptamer molecular weight of about 18.6 kDa, we obtain the surface density of the nucleic acid, equivalent to about 4.8 × 10^12^ molecules per cm^2^. This result is very similar to those that can be found in the literature for an aptamer monolayer specifically bonded to a surface [26].

The changes in frequency and dissipation following the addition of the *S. aureus* at concentration 10^7^ CFU/mL are 139.6 ± 22.6 Hz and (13.2 ± 3.7) × 10^−6^, respectively. Then, the surface mass density of bacteria can be estimated as (0.96± 0.16) µg∙cm^−2^. Considering that *S. aureus* has a spherical shape of a diameter approximately 0.5 µm, and that the density of the cytoplasm is approximately 1 g∙cm^3^ [27], one can estimate that the average mass of one bacterium is 0.065 pg. Therefore, the surface density of the bacteria can be estimated as 1.48 × 10^7^ bacteria∙cm^−2^. Considering that the cross-sectional area of one bacterium is approximately 0.2 µm^2^, at full coverage, the surface density will be 5 × 10^8^ bacteria∙cm^−2^, which is much higher in comparison with those estimated from frequency changes. However, considering that *S. aureus* forms colonies, the surface density can be lower in comparison with those estimated from frequency changes. It is also evident that the surface density of bacteria is much lower than those of the aptamers. This means that a large number of aptamers bind to one bacterium.

In the literature, there have been a limited number of aptasensors for *S. aureus* explored. Table 2 summarizes some of the most sensitive aptasensors, which have LODs that are similar to this work’s TSM-based aptasensor. However, some of the aptasensors were tested in culture, which is less fouling than whole milk. Our developed TSM aptasensor has excellent sensitivity that is better or comparable to other reported aptasensors. Moreover, our sensor has the advantage of operating in raw milk due to its antifouling thiol linker, DTT_COOH_, making detection rapid and efficient for the analysis of milk.

### 3.4. Effect of Aptamer/Ethanolamine Immobilization and Bacteria Binding on the Viscoelastic Properties of the Sensing Layers

It is possible to estimate the mass of aptamer immobilized on the electrode with the Sauerbrey equation, as well as ethanolamine (used to deactivate the activated carboxyl groups of the SAM and increase the antifouling characteristics of the surface). The mass of aptamer was found to be 30.0 ± 5.1 ng, while ethanolamine was 4.5 ± 2.9 ng. These values were then used for the subsequent calculations.

We also analyzed changes in the viscosity coefficient, *η*, shear modulus, *μ*, and penetration depth, *Γ*, following incubation of the DTT_COOH_ SAM with aptamer, ethanolamine, and bacteria. Since it was not possible to measure the viscoelastic properties of the thiol layer (the functionalization of the thiols was conducted in vial, not in a flow), the values of the viscoelastic parameters are relative and not absolute. The viscoelastic parameters are shown in Table 3. The analyses were performed on three independent measurements and the data are represented as mean and standard deviation.

The variation of the shear modulus, µ, for the aptamer layer has a magnitude that is comparable with biomolecules, and certainly smaller than the quartz crystal (~10^10^ Pa) [37]. Regarding the viscosity coefficient, *η*, this was lower than compact protein layers such as those obtained with β-casein (~10^−4^ Pa∙s). This may suggest that the aptamer layer is not quite compact, but rather that the nucleic acid tends to have a loose, potentially globular three-dimensional structure which does not allow for high density packing. This would agree with the idea that aptamers need to fold in order to perform their receptor function. The penetration depth, *Γ*, of the mechanical evanescent wave is around 200 nm for a crystal with a fundamental frequency of 8 MHz [38]. This value varies substantially little following the formation of a compact SAM, while by immobilizing polymeric molecules, one should see a significant reduction. In fact, following the immobilization of the aptamer, the penetration depth is reduced by about 1/6, emphasizing how effectively the aptamer is present on the layer. However, due to its steric characteristics, the aptamer does not pack to form a dense layer. This is an important prerequisite for the formation of an aptasensor as the aptamer has greater freedom to rearrange itself in the presence of the specific analyte and bind more effectively to it.

The variation of the shear modulus for ethanolamine is less than that obtained following the incubation of the aptamer, meaning that ethanolamine is precisely bound to the SAM, contributing to the formation of a dissipative and non-elastic mix layer. However, being a small molecule, it has less influence on the elastic properties. In the case of the viscosity coefficient, *η*, this is also varied to a very small extent, indicating that this small molecule has very little influence on the density characteristics of the functionalized layer. However, as one would expect, the coefficient of viscosity increases slightly (the mix layer, with the contribution of ethanolamine, becomes more viscous). The penetration depth changed very little compared to the aptamer, confirming that it is precisely the ethanolamine that binds to the activated carboxyl groups of DTT_COOH_, which, being a small molecule, has little influence on the path of the evanescent acoustic wave. Additionally, the small variation in penetration depth also demonstrates that ethanolamine molecules bind distantly from each other (i.e., only where the DTT_COOH_ tails are) and therefore not densely.

Following the incubation of the sensing layer with bacterial cells, the shear modulus increases to values greater than those of aptamer/ethanolamine alone, while the viscosity coefficient decreases. It is likely that the bacterial cell, by binding to the free end of the aptamers, reduces the movement of the latter, causing the formation of a more tightly packed layer and, consequently, one which is more elastic and slightly less dissipative (hence the increase in the shear modulus and the reduction of the coefficient of viscosity). However, the penetration depth does not vary significantly, increasing only slightly compared to the aptamer/ethanolamine heterolayer, since the acoustic wave propagates better in the presence of a more compact structure.

### 3.5. Specificity of Staphylococcus aureus Detection

**Figure 7:**
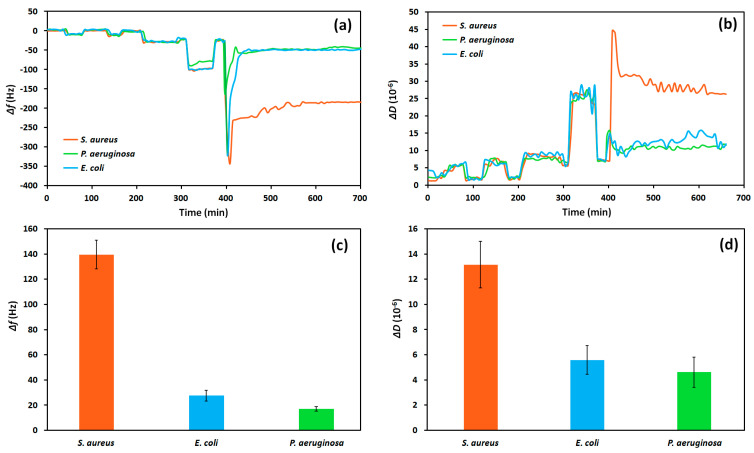
The (**a**) frequency, Δ*f*, shift, the (**b**) dissipation, Δ*D*, shift, the (**c**) overall frequency change, and the (**d**) overall dissipation change as a result of exposing aptasensors to whole milk spiked with 10^7^ CFU/mL of *S. aureus*, *P. aeruginosa*, and *E. coli*.

To determine if the developed aptasensor is specific to *S. aureus*, it was also tested against 10^7^ CFU/mL of *P. aeruginosa* and *E. coli* in whole milk. The frequency and dissipation were measured for each (Figure 7). We used relatively high concentrations of bacteria to be sure that no binding of bacteria other than *S. aureus* occurs, even at high concentrations. 

The overall change in both frequency and dissipation are much higher for the aptasensor in response to *S. aureus* compared to the other bacteria. With *S. aureus*, the overall frequency variation was approximately 140 Hz, while *E. coli* was 27 Hz, and *P. aeruginosa* was only 16 Hz, representing an 80% and 89% greater signal for *S. aureus* compared to these other bacteria, respectively. Though some frequency change is observed for the other bacteria (due to a minimal fouling of milk), the much stronger signal for *S. aureus* shows that this sensor is still quite specific to the target bacteria and can be used to determine the specific contamination of samples caused by *S. aureus*. 

## 4. Conclusions

A thickness shear mode (TSM) acoustic aptasensor was developed for detecting *Staphylococcus aureus* in whole UHT cow’s milk. Detection was achieved without treating the milk due to the antifouling layer of the aptasensor, which employs the thiol linker 3-dithiothreitol propanoic acid (DTT_COOH_). We have shown that after linker synthesis, Fourier transform infrared spectroscopy (FTIR) can be used to analyze and confirm the structure of the desired molecule. Once DTT_COOH_ was linked to the aptamer, the antifouling character of the sensor significantly improved. Relative to the DTT_COOH_ layer, the DTT_COOH_-aptamer layer experienced less milk fouling by approximately 88% and the frequency and dissipation variations were minimal in whole milk.

We used the frequency variation and dissipation data obtained during the construction of the aptasensor to demonstrate the correct immobilization of the components. In particular, the DNA aptamers bind to the surface in a non-dense manner, which favors recognition and binding with the analyte. Ethanolamine plays a marginal role in the variation of the viscoelastic parameters, which was expected, as it is a small molecule. However, the binding of bacteria to the sensing surface resulted in an increasing shear modulus, evidencing the reduction in the mobility of the aptamer layers. 

The developed aptasensor achieved excellent sensitivity and specificity and was able to successfully detect and quantify *S. aureus* in milk. As the 33 CFU/mL limit of detection and 101 CFU/mL limit of quantification are significantly below the EU’s safe limit for bacteria in milk products, the aptasensor is practical for rapid and sensitive detection in the milk industry. Additionally, the sensor was found to be specific to *S. aureus* compared to other tested bacteria. However, to make TSM more user-friendly, its design requires further engineering. The TSM aptasensor developed in this work demonstrates that a sensor based on the novel antifouling linker DTT_COOH_ can rapidly detect and quantify *S. aureus* directly in raw whole UHT milk samples. Further work will explore the use of this sensor for different bacteria using their unique aptamers.

## Figures and Tables

**Table 1:** Frequency, Δ*f*, and dissipation, Δ*D*, changes after exposure to whole milk and washing with running buffer. % fouling relative to DTT_COOH_ has been calculated as (Δ*f*/Δ*f*_DTTCOOH_)x100, where Δ*f* denotes the frequency changes for corresponding SAM surface. Average and standard deviations (SD) were determined from three independent experiments.

SAM	Δ*f* (Hz)	%Fouling Relative to DTT_COOH_	Δ*D* (10^−6^)
UDT	158 ± 16	64	8.0 ± 2.4
MUA	136 ± 4	58	7.6 ± 0.8
Bare gold	105 ± 5	45	6.1 ± 0.6
DTT	40 ± 8	−31	2.8 ± 1.4
DTT_COOH_	58 ± 14	-	3.4 ± 2.7
Aptasensor	7 ± 1	−88	0.9 ± 0.2

**Table 2:** An overview of aptasensors used for the detection of *S. aureus*.

*S. aureus* Strain	Material Platform	Method of Detection	LOD, CFU/mL	Linear Range, CFU/mL	Sample	Reference
*Acoustic aptasensors*						
KR3	Gold-thiol-NH_2_-Aptamer	TSM	33	10^2^–10^6^	Milk	This work
ATCC 25923	Aptamer/graphene gold IDE	IDE-SPQC	41	41–4.1 × 10^5^	Culture	[28]
ATCC 25923	Aptamer/graphene gold IDE	IDE-SPQC	41	41–4.1 × 10^5^	Milk	[28]
N/A	Avidin-biotinylated aptamer	Magnetostriction	5	10–10^11^	Water	[29]
*Electrochemical aptasensor*						
ATCC 25923	Aptamer-conjugated AgNPs	DPV	1	10–10^6^	Culture	[30]
*Optical aptasensors*						
N/A	Aptamer-conjugated MGNPs	SERS	35	10^2^–10^7^	Culture	[31]
04018	AgMNPs	SERS	10	10–10^5^	Culture	[32]
ATCC 29213	Aptamer-functionalized graphene oxide	Fluorescence	800	10^3^–10^6^	Culture	[33]
N/A	Aptamer-fluorescent silica nanoparticles	Fluorescence	760	-	Milk	[34]
ATCC 2921	Biotinylated aptamer-streptavidin	Colorimetric	9	10–10^6^	Milk	[35]
ATCC 29213	Aptamer-UCNPs	Fluorescence	25	50–10^6^	Culture	[36]

DPV: differential pulse voltammetry; AgNPs: silver nanoparticles; MGNPs: Fe_3_O_4_ magnetic gold nanoparticles; AgMNPs: Ag-coated magnetic nanoparticles; IDE-SPQC: interdigital electrodes-series electrode piezoelectric quartz crystal; UCNPs: upconversion nanoparticles.

**Table 3:** Variation of viscoelastic properties following DNA aptamer and ethanolamine incubation as well as at presence of highest concentration of *S. aureus* (10^7^ CFU/mL). Results are mean ± S.D. obtained from 3 independent experiments.

Viscoelastic Parameter	DNA Aptamer	Ethanolamine	*S. aureus* at 10^7^ CFU/mL
μ (10^4^ Pa)	4.01 ± 0.24	1.6 ± 0.3	6.03 ± 0.31
*η* (10^−5^ Pa∙s)	6.01 ± 0.61	6.04 ± 0.63	3.84 ± 0.14
* Γ * (nm)	33.7 ± 4. 9	27.7 ± 4.5	29.1 ± 0.1

## Data Availability

Not applicable.

## Supplementary Materials

The following supporting information can be downloaded at: https://www.mdpi.com/article/10.3390/bios13060614/s1, Section S1: FTIR Analysis of DTT_COOH_, Section S2: Secondary structure of DNA Aptamer, Section S3: CFU Counts of *Staphylococcus aureus*, Section S4: Experimental Setup.
