# Supplementary material for: Staphylococcus aureus Detection in Milk Using a Thickness Shear Mode Acoustic Aptasensor with an Antifouling Probe Linker
Source: Biosensors (Basel). 2023 Jun 3;13(6):614. doi: 10.3390/bios13060614 (PMC10296631; doi:10.3390/bios13060614)
Supplement: Supplementary file 1 [file biosensors-13-00614-s001.zip › biosensors-2419672-supplementary.pdf]

biosensors

MDPI

Supplementary Material

Staphylococcus Aureus Detection in Milk Using a Thickness Shear Mode Acoustic Aptasensor with an Antifouling Probe Linker

Sandro Spagnolo [1], Katharina Davoudian [2], Brian De La Franier [2], Tibor Hianik [1,*], and Michael Thompson [2,*]

[1] Faculty of Mathematics, Physics and Informatics, Comenius University, Mlynská dolina F1, 842 48 Bratislava, Slovakia

[2] Department of Chemistry, University of Toronto, 80 St. George Street, Toronto, ON M5S, Canada

* Correspondence: tibor.hianik@fmph.uniba.sk (T.H.) and m.thompson@utoronto.ca (M.T.)

S1. FTIR Analysis of DTT_{COOH}

Analysis of DTT_{COOH} by Fourier transform infrared spectroscopy (FTIR) was performed using a Prestige-21 FTIR spectrometer (Shimadzu, Kyoto, Japan). The measurement was carried out in transmittance mode using CaF_2 cell (Specac, Orpington, UK). DTT_{COOH} was solubilized in ethanol at a concentration of 4 mM. Subsequently, drops of the sample were poured onto the cell and the measurement was conducted before and after solvent evaporation.

The FTIR spectra for DTT_{COOH} is shown in Figure S1. C-H and S-H bond stretches are expected from 3000-2700 cm^{-1}. However, DTT_{COOH} absorbs infrared broadly due to numerous O-H bonds.

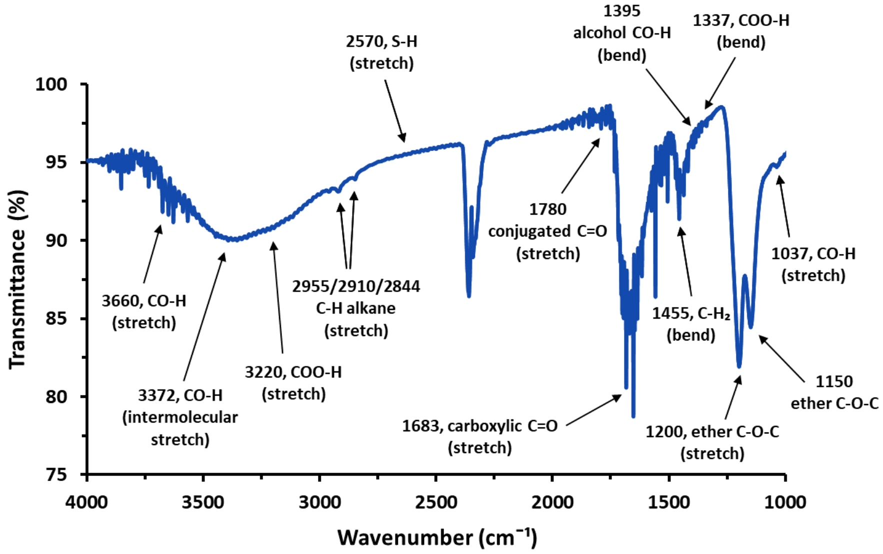

Figure S1. FTIR transmittance spectrum of DTT_{COOH} following solvent evaporation.

To reduce the broad absorption of O-H stretches, further analysis was carried out in solution (Figure S2), allowing for the solvent spectrum to be subtracted.

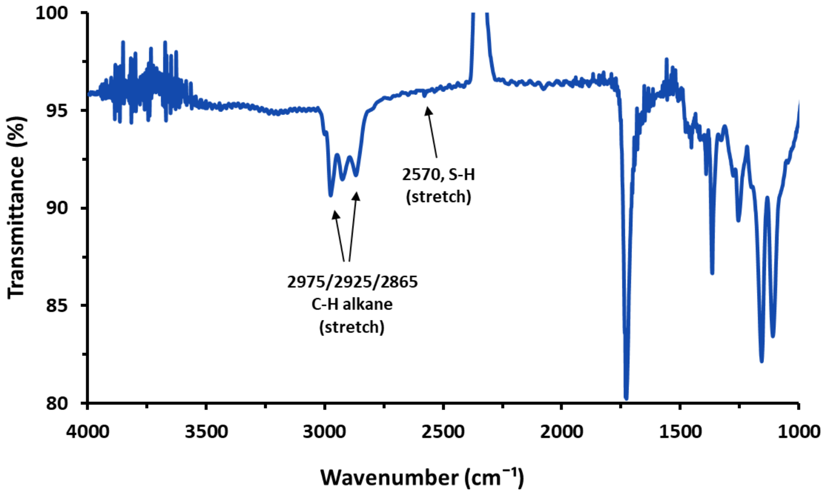

Figure S2. FTIR spectrum of DTT_{COOH} in a solution of ethanol. C-H and S-H bond stretches are more noticeable in the region of 3000-2700 cm^{-1}.

S2. Secondary Structure of DNA Aptamer

We applied OligoAnalyzer Tool™ (Integrated DNA Technologies, Inc., Coralville, IO, USA) program for analysis of possible secondary structure of the aptamer (5′ NH_2-TCC CTA CGG CGC TAA CCT CCC AAC CGC TCC ACC CTG CCT CCG CCT CGC CAC CGT GCT ACA AC-3′). According to this program there exists 9 variations of the secondary structure. Two of them that are characterized by lowest Gibbs energy, and consequently most stable: - 12.2 kJ/mol (A) and -11.8 kJ/mol (B), respectively, are presented on Figure S3.

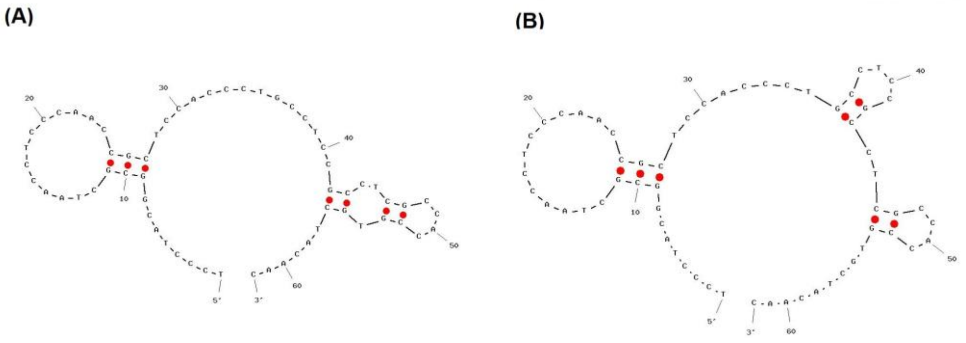

Figure S3. Possible secondary structure of DNA aptamers used in this work generated by OligoAnalyzer Tool™ that differ by Gibbs energy: - 12.2 kJ/mol (A) and -11.8 kJ/mol (B).

S3. CFU Counts of *Staphylococcus Aureus*

To quantify the amount of *S. aureus* being used in experiments optical density at 600 nm was used. To correctly determine the bacterial concentration using OD600, CFU counts at multiple dilutions of *S. aureus* were collected and compared to the OD600 at each dilution (Figure S4).

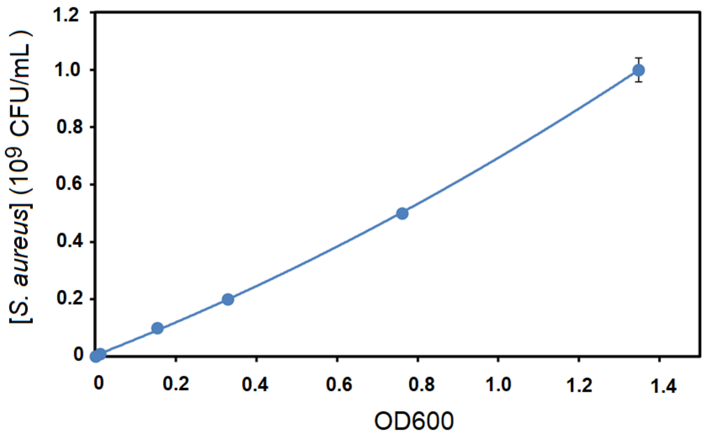

Figure S4. Concentration of *S. aureus* in PBS compared to their optical density at 600 nm (OD600).

A polynomial relationship between the OD600 measurements and CFU counts for *S. aureus* in PBS was found with a relationship of:

$$[S.\ aureus]\ (\mathrm{CFU/mL}) = 1\times10^{8}\ x^{2} + 5\times10^{8} + 8\times10^{6}$$

where x is the OD600 value. Using this relationship the grown solutions of *S. aureus* could be diluted to the desired concentration for measurement.

S4. Experimental Setup

Figure S5 shows the scheme of experimental setup consisting of an Acryl flow cell with a volume 100 µL, syringe pump and vector analyzer.

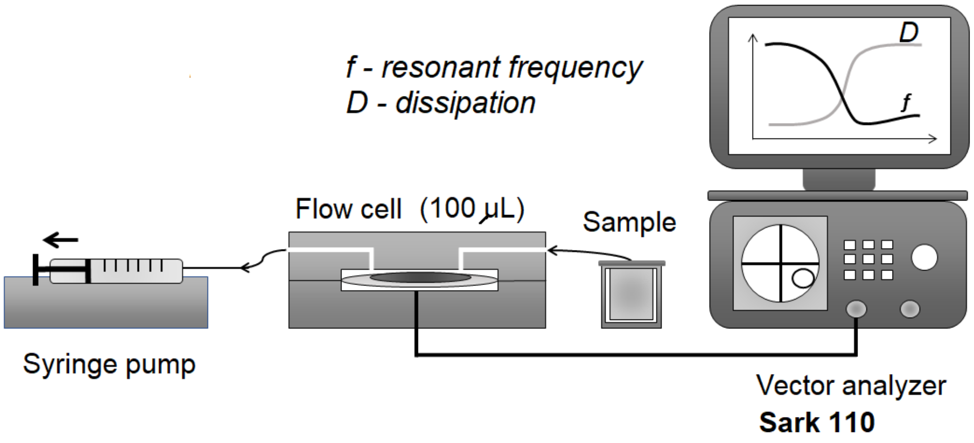

Figure S5. The scheme of experimental setup.
